# Evolution and Comparative Analysis of Clinical Trials on Psilocybin in the Treatment of Psychopathologies: Trends in the EU and the US

**DOI:** 10.3390/jcm14186613

**Published:** 2025-09-19

**Authors:** Anastasia Calin, Ana Flavia Burlec, Cornelia Mircea, Irina Macovei, Monica Hancianu, Andreia Corciova

**Affiliations:** 1 Department of Drug Analysis, Faculty of Pharmacy, Grigore T. Popa University of Medicine and Pharmacy, 16 University Street, 700115 Iasi, Romania; anastasia_calin@email.umfiasi.ro (A.C.); irina-macovei@umfiasi.ro (I.M.); maria.corciova@umfiasi.ro (A.C.); 2 Department of Pharmaceutical Biochemistry and Clinical Laboratory, Faculty of Pharmacy, Grigore T. Popa University of Medicine and Pharmacy, 16 University Street, 700115 Iasi, Romania; cornelia.mircea@umfiasi.ro; 3 Department of Pharmacognosy, Faculty of Pharmacy, Grigore T. Popa University of Medicine and Pharmacy, 16 University Street, 700115 Iasi, Romania; monica.hancianu@umfiasi.ro

**Keywords:** psilocybin, clinical trials, psychopathologies, psychedelics, regulatory policies

## Abstract

**Background/Objectives:** This study examines the development of clinical trials investigating psilocybin for the treatment of psychopathologies, with a comparative focus on the United States (US) and the European Union (EU). The objective is to identify regional differences in trial progression, research infrastructure, and regulatory frameworks. **Methods**: A mixed-methods approach was applied, combining case studies, qualitative and quantitative research. Key variables included trial phase, geographical distribution, demographic factors, funding, governmental support, and public health policies. **Results**: The US demonstrated a substantially higher number of psilocybin trials across both early and advanced phases. This reflects a strong research infrastructure, growing financial investment, and increasing interest in psychedelic-assisted therapies. In contrast, the EU showed fewer trials and slower advancement, reflecting a more cautious stance that emphasizes patient safety and therapeutic efficacy. These divergences are shaped by differences in regulation, funding mechanisms, and sociocultural attitudes toward psychedelics in psychiatry. **Conclusion**: This comparative analysis highlights the uneven pace of psilocybin research across different regions. It also emphasizes the importance of international collaboration, harmonization of public health policies, and the development of standardized procedures prioritizing safety and effectiveness. Integrating psilocybin-assisted interventions into psychiatric practice has the potential to expand treatment options and strengthen mental health care, but coordinated global efforts are essential to ensure both scientific rigor and patient protection.

## 1. Introduction

Over the past two decades, under pressures arising mainly from the apparent limitations of current therapies for mental disorders, the international scientific community has begun to re-evaluate the therapeutic potential of previously stigmatised substances. Among these substances is psilocybin, a naturally occurring organic compound isolated from hallucinogenic mushroom species belonging to the genera *Psilocybe, Panaeolus*, and *Gymnopilus.* Psilocybin has caught the attention of researchers due to its distinctive pharmacological profile and therapeutic potential, with emerging prospects for complementary treatment in clinical medicine [1,2].

Throughout history, mushrooms containing psilocybin have been used in traditional medicine, playing a fundamental role in both curative and spiritual practices in many cultures [3]. In particular, mushrooms of the genus *Psilocybe* were used in the spiritual ceremonies and healing practices of the Aztec and Mazatec civilisations, and were considered sacred [2].

The scientific rediscovery of this substance and its effects took place thousands of miles away from its original use, at Sandoz Laboratories in Basel, Switzerland, by Albert Hofmann in 1958. The period between 1958 and 1960 marked the beginning of research into the pharmacological effects of psychedelic substances, reaching its clinical peak in 1960 when psilocybin was used for the first time in a clinical study.

The public perception of psychedelic substances, especially psilocybin, underwent a radical change in the 1970s. The President of the United States (US) at that time, Richard Nixon, approved the Controlled Substances Act (Section 812) in 1971, which classified psilocybin in the Schedule I table, an official list of illegal substances, considering it to be among the most dangerous. Its inclusion in this schedule highlighted the high risk of developing addiction to these substances, while also minimising their curative potential [3].

Over the past three decades, the scientific stigma surrounding psilocybin has gradually faded, and its reputation has been restored through growing recognition of its scientific and therapeutic value—now evident in the ethical and institutional approvals granted in both the United States and European Union member states. These allow the pharmacological and curative effects of psilocybin to be studied in detail in advanced clinical trials conducted at prestigious academic institutions [2,4]. Such institutions include Johns Hopkins Centre for Psychedelic and Consciousness Research, NYU Langone Health—Centre for Psychedelic Medicine, Massachusetts General Hospital—Centre for the Neuroscience of Psychedelics (US), King’s College London—Psychoactive Trials Group and Imperial College London—Centre for Psychedelic Research (UK), University Medical Centre Groningen (The Netherlands), Fundação Champalimaud (Portugal), National Institute of Mental Health (Czech Republic), University of Copenhagen and Bispebjerg Hospital (Denmark) and Centrum Badań Klinicznych PI-House (Gdańsk) (Poland). As a result, there is a growing trend toward integrating psilocybin into clinical research and exploring the implications of its therapeutic potential in both psychiatric and neurological disorders.

A significant milestone in establishing the research trajectory for this substance of interest was the 2019 decision by the FDA (Food and Drug Administration) to grant psilocybin “Breakthrough Therapy” status based on results observed in clinical trials investigating its use as a treatment for treatment-resistant depression [5]. Evidence from these rigorous clinical trials has shown that psilocybin produces long-lasting (6–12 months) antidepressant effects after a single administration when accompanied by specialised psychotherapeutic support. At the same time, no risks of abuse or central toxicity were reported throughout the clinical trials or during the post-study observation period (1–3 years) [6,7].

Therefore, psilocybin is an important pillar of the broad movement to reconceptualise how mental and neurological disorders are treated. This movement emphasises improving patients’ quality of life, not only by alleviating symptoms, but also by facilitating individuals’ reconnection with their existence and changing their perception of their own lives [1].

The overall objective of this paper is to compare how public health policies in the EU and the US integrate clinical research and the use of psilocybin and its curative potential. At the same time, it begins with the assumption that the process and timing of developing and adopting legislation on clinical trials involving psilocybin vary regionally, depending on the legal, cultural, and scientific context of the region concerned. Thus, this paper considers the following questions:

1. How do clinical trial trends involving psilocybin differ between the US and the EU?
2. How do regulatory policies address psilocybin’s potential medical use?
3. What are the observed differences in public and medical perceptions across the two regions?

The comparison between the two regions of interest is justified not only by the influence that drug and clinical trial regulatory institutions have on global health policies, but also by the specificity of the regulatory and funding systems for medical and pharmaceutical research [2,8].

## 2. Materials and Methods

The methodological approach of this paper is mixed, given that it addresses both qualitative and quantitative components.

### 2.1. Quantitative Methods

Data sources & date cut-off: ClinicalTrials.gov and the EU Clinical Trials Information System (CTIS) were queried on 31 May 2025 for trials listing psilocybin or psilocin in the intervention field. Interventional studies of any phase with therapeutic intent were included; phase 0 defined as microdosing and early pharmacokinetic characterization.

Inclusion criteria: interventional design; adult or paediatric populations with psychiatric/neurologic indications; any recruitment status (recruiting/active/completed/suspended/terminated/unknown).

Exclusion criteria: purely mechanistic imaging without clinical endpoints; duplicate postings; non-psilocybin psychedelics unless explicitly combined with psilocybin.

Cross-registry reconciliation and multicenter handling: Trials were counted by unique registry identifier within each registry (NCT for ClinicalTrials.gov; CTIS/EudraCT for CTIS). No cross-registry deduplication was performed; consequently, dual-registered studies may appear in both registry-specific aggregates. Multicenter and multiregional trials (e.g., sites in both the United States and the European Union) were treated as a single trial in aggregate counts. Geographic summaries reflect registry-reported location fields and indicate country/state appearances rather than additional unique trials; site-level apportionment was not performed.

Participant total estimation: A single estimated participant total was obtained by summing the registry Enrollment field across included records, using Actual Enrollment were reported and Estimated/Anticipated Enrollment otherwise. Records lacking a numeric enrollment value were excluded from the sum. Because cross-registry deduplication was not performed and enrollment reporting varies by registry and study status, the combined participant total should be interpreted as approximate.

Abstraction & verification: Data were abstracted by a single reviewer using a structured extraction template with automated checks (identifier-format validation, controlled vocabularies for phase/status, and enrollment range checks). No independent verification was performed; consequently, residual errors or omissions may be present and should be considered in interpretation.

Data availability: Underlying datasets (master trial table and figure datasets) can be made available upon reasonable request.

### 2.2. Qualitative Methods

The qualitative component of this study involves the analysis of specialised literature, review studies, and specialised documents involving regulations in this field, identified through the use of recognised databases such as PubMed, Elsevier, Springer, Web of Science, as well as the official websites of relevant organisations such as the FDA, EMA, and EUDA (European Union Drugs Agency). The qualitative analysis focused on the following components: justification of the therapeutic use of psilocybin, mechanisms of action, context of clinical studies, regulatory mechanisms, and integration of the substance of interest into public health policies, as well as their ethical and clinical implications. The quantitative analysis involved extracting and systematising data from official clinical trial registries: ClinicalTrials.gov for clinical trials conducted in the US and adjacent regions, the EU Clinical Trials Information System (CTIS, successor to EU-CTR) for trials authorised in EU member states, and PubMed for identifying publications associated with the identified trials [9].

The clinical studies analysed were divided into two groups: completed and ongoing. The following characteristics were collected for each study: clinical development phase, geographical location, sponsor, sample size, age of volunteers, type of study (randomised or non-randomised, open or closed, multicentre or single-centred), and estimated duration of observation. For completed studies, the management of adverse reactions and the post-study observation period were also analysed.

This investigation takes an exploratory approach, given the novel and complex nature of the research field, in which both theoretical concepts and the clinical framework are still evolving. The comparative dimension of this study allows the identification of significant trends, discrepancies, and similarities between the US and the EU regarding the regulatory framework and the future direction of public health policies in which these therapies may be integrated [10,11].

## 3. Psilocybin and Its Therapeutic Potential in Mental Disorders

### 3.1. Mental Disorders Investigated in Psilocybin Studies

Over the past two decades, psilocybin and other substances in the psychedelic class have entered a new phase of scientific exploration, being investigated for their therapeutic potential in treating a wide range of mental disorders. Among the most common conditions studied in research proposing psilocybin as a therapy are major depression (MD), recurrent depressive disorder (RDD), anxiety disorders (AD), post-traumatic stress disorder (PTSD), substance use disorders and addictions, and obsessive-compulsive disorders (OCD) [12,13,14].

Data provided by the World Health Organisation (WHO) show that mental disorders affect over 970 million people globally, representing 13% of the world’s population [15].

Of the conditions listed above, depression is by far one of the most common, often diagnosed in association with other disorders, such as behavioural and anxiety disorders. Their high prevalence is also associated with social, economic, and family instability in patients. The quality of life of those diagnosed, as well as that of their loved ones, is often severely affected by the deterioration of family and interpersonal relationships, as well as social and professional activity [15,16].

Untreated or treatment-resistant depression is frequently associated with behavioural dysfunctions, resulting in the inability of those affected to perform tasks related to social and parental roles [17]. The pathological mechanism of severe depression involves a dysfunction of the serotonergic system, reduced activity in the dorsolateral prefrontal cortex, and hyperactivity in the cerebral amygdala, which contributes to mood disorders, cognitive distortions, and rumination. In addition, hypothalamic–pituitary–adrenal axis dysregulation and neurobiological inflammation play a significant role in the pathophysiology of treatment-resistant depression [18].

In this context, current research is focusing on studying the effects of psilocybin as a treatment for severe forms of depression or refractory forms of this disorder. Recent data from studies in which psilocybin is administered in a single dose in a controlled environment and is combined with psychotherapy indicate promising results in patients affected by TRD. Post-administration observations have shown a decrease in symptom severity and suggest a rapid mechanism that differs from that of classical antidepressants [14,19,20].

PTSD is associated with maladaptive consolidation of traumatic memory, accompanied by amygdala hyperactivity, medial prefrontal cortex hypofunction, and a structurally reduced hippocampus. These changes contribute to exaggerated stress responses and deficits in emotional regulation and fear inhibition [21]. In studies focusing on the treatment of PTSD, psilocybin is being investigated for its ability to adaptively reconsolidate traumatic memory, reduce stress on neural networks, and reduce their hyperactivity, which is created and stimulated by primary stress conditions [22]. Although studies in this direction are in their early stages, the results obtained for psilocybin on the modulation of neural tissues in studies conducted on other conditions are encouraging.

Anxiety disorders (AD) are characterised by hyperactivity in neural circuits involved in danger anticipation, particularly in the amygdala and thalamocortical networks. Dysfunction of the GABAergic and serotonergic systems contributes to the maintenance of hypervigilance and catastrophic interpretation of neutral or ambiguous stimuli [23]. Multidisciplinary and multicentre research has been conducted to analyse the potential of psilocybin as a treatment for alleviating symptoms associated with AD, particularly those related to terminal somatic illnesses. These studies have shown a rapid reduction in anxiety and depressive symptoms with improvements in social and relational functioning [8,24,25]. At the same time, it has been found that in these cases, psilocybin provides not only symptomatic relief but also a profound existential change. The treatment of severe anxiety disorders, particularly those associated with a poor cancer prognosis, is being studied at university medical research centres such as New York University and Johns Hopkins [26].

An emerging subfield of research in which psilocybin has started to be explored is that of eating disorders, particularly anorexia nervosa. Anorexia nervosa involves persistent neurobiological abnormalities in dopaminergic reward circuits and cortical regions involved in self-evaluation, such as the anterior cingulate cortex. This cognitive rigidity and aversion to food are supported by altered functional connectivity and increased sensitivity to internal signals related to control and order [27]. This condition is characterised by severe self-imposed food restriction, body image distortion, and an increased mortality rate [16].

Another subdomain of mental illness for which research involving psilocybin has been conducted is substance use disorders (addictions). These disorders are characterised by a persistent disruption of the brain circuits involved in the biological mechanisms of reward, motivation, and behavioural control, particularly in the mesolimbic dopaminergic system. Repeated administration of substances with a high risk of dependence causes hyperactivity of the dopaminergic pathway between the ventral tegmental area and the nucleus accumbens, which accentuates abusive behaviour through conditioned learning mechanisms. At the same time, there is also an inhibition of the dorsolateral prefrontal cortex’s function, which is involved in inhibitory control, decision-making, and the regulation of compulsive impulses, leading in the long term to a distortion of reality, a distorted self-image, and a loss of control over consumption [28].

In this context, psilocybin has been investigated for its potential to modulate neural connections and implicitly disrupt circuits disrupted by substance abuse. Studies have shown that psilocybin treatments have led to reduced cognitive rigidity and promoted a type of self-reflection on one’s compulsive behaviours, described by patients as a “mental reset.” A pilot study conducted by Bogenschutz et al. in 2015 showed that psilocybin administered in combination with motivational therapy significantly reduced alcohol consumption in patients diagnosed with severe alcoholism [29]. These results were confirmed by a comparative analysis with control groups where the abstinence rate was up to 30% lower over a 6-month observation period. A similar result was reported in a study coordinated by Johnson et al. in 2014 at Johns Hopkins University, where smoking patients received psilocybin treatment [30]. The analysis showed that 80% of smokers who received psilocybin remained abstinent for 6 months, compared to only 35% in the group that received conventional treatments.

### 3.2. Psilocybin—Origin, Mechanisms of Action, Effects

Psilocybin is a naturally occurring psychoactive substance belonging to the alkaloid class, also known as 4-phosphoryloxy-N, N-dimethyltryptamine.

It is produced by several species of fungi, particularly from the genera *Psilocybe*, *Panaeolus*, *Gymnopilus*, and *Copelandia*, which are widespread in tropical and subtropical regions. Additionally, 180 other species of fungi are known to produce psilocybin, albeit in smaller quantities [2]. Chemically, psilocybin is a prodrug, as it is rapidly dephosphorylated in the gastrointestinal tract by the enzyme alkaline phosphatase after ingestion and converted into the biologically active compound psilocin [8].

The use of psilocybin was documented by Spanish chroniclers and colonists in South America in the 16th century. Mesoamerican peoples used mushrooms with high psilocybin content in rituals and ceremonies, as well as a form of treatment for a wide range of conditions and symptoms, ranging from various types of pain to insomnia. Although considered a fundamental part of Aztec and Mazatec cultures, initiation rituals and the use of mushrooms to treat the sick were restricted and marginalised by new colonial rules, being considered by the Spanish to be blasphemy against Christianity [2].

Psilocybin was rediscovered in the West in the 1950s through the expedition of ethnobotanist Gordon Wasson to Mexico. He participated in initiation and reconnection rituals, which are preserved in the cultures of the Mazatec and Nahua peoples and practised in the context of spiritual healing and shamanic initiation. Psilocybin was subsequently isolated and synthesised by Albert Hofmann in 1958, the same researcher who synthesised LSD [30,31]. Although psilocybin was used in experimental psychiatric research between 1959 and 1965, it was banned under the anti-drug regulations of the 1970s, starting with the constitutional act approved in 1971, the Controlled Substances Act, Section 812(a) [2,8]. At European level, the legislative context surrounding psilocybin was significantly influenced by global politics and prevailing medical paradigms. Based on the aforementioned constitutional act, the United Nations (UN) included psilocybin on the list of prohibited, dangerous, and therapeutically insignificant substances in the 1971 Convention on Psychotropic Substances. Following this move, most European countries synchronised their legislation on psychedelic substances, thereby prohibiting the production, sale, and possession of such substances.

**Figure 1:**
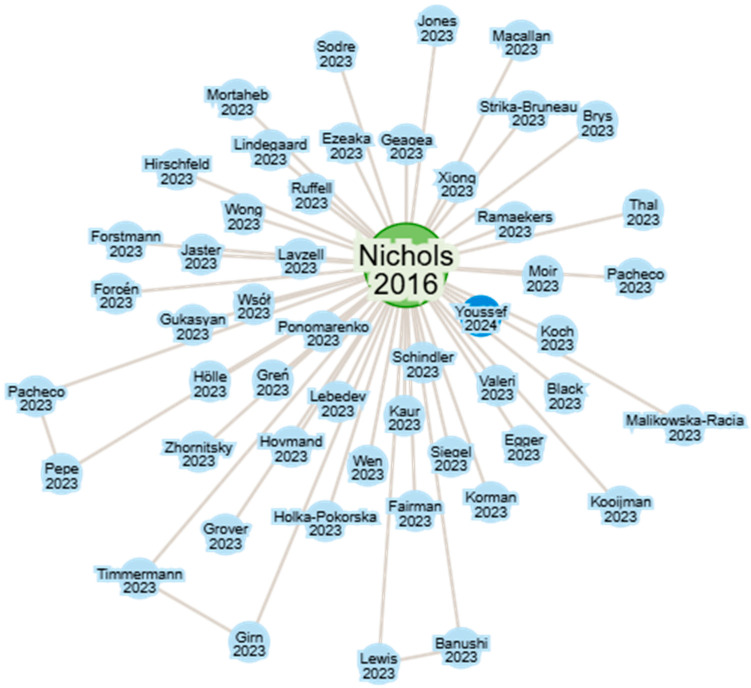
Network of articles related to the research published by Nichols et al., 2016 [9]. Source: ResearchRabbit.ai. Description: Automatically generated visualisation based on David E. Nichols’ article (2016), showing recent works that directly cite it.

The review article published by David E. Nichols in 2016 was a turning point in the reopening of scientific research on psychedelic substances, particularly psilocybin. Figure 1 illustrates the article’s impact in the scientific literature, highlighting the significant number of studies that cite it after 2020, which confirms a resurgence of interest in this molecule [9].

Although legislation on psilocybin is not yet fully harmonised in the US and the EU, with psilocybin remaining criminalised in most states, there is nevertheless a trend toward authorising it through special regulations for scientific research. Countries such as Germany, Denmark, Portugal, and the Netherlands have implemented new legislation in recent years that supports clinical research on psychedelic substances, signalling a shift in perception at the government level [32].

In contrast, in Romania, psilocybin is classified as a prohibited substance under Law No. 143/2000 on the prevention and combating of illicit drug trafficking and consumption, as amended and supplemented. This substance is included in Schedule I, being defined by this classification as a substance with a high potential for abuse and no known therapeutic effects or recognised medical use [33]. However, according to the law that includes psilocybin in Schedule I of classified substances in Romania, an exception is granted for research activities, provided that the dose used does not exceed the approval threshold and that the methodology applied is subject to rigorous analysis, by Law No. 339/2005, as amended and supplemented. To date, no clinical studies have been authorised to investigate the therapeutic effects of psilocybin in Romania, and our country has not been included as a member in multicentre research projects conducted at the European level [34].

**Figure 2:**
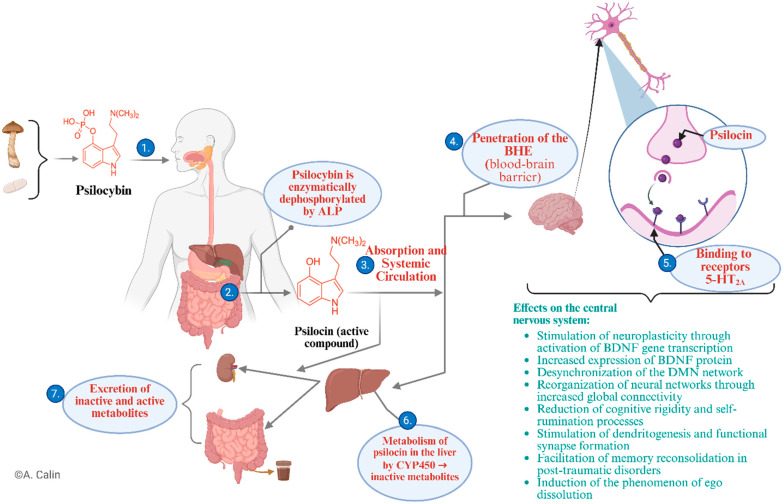
Pharmacokinetic profile and mechanism of action of psilocybin in the human body. Source: Created in Biorender. Anastasia, C. (2025) https://app.biorender.com/illustrations/682c438794135305917fad3a?slideId=bb8edc16-f078-4768-8d01-199416da2336.

From a biological point of view, psilocybin acts predominantly through its active metabolite, psilocin, which crosses the blood–brain barrier (BBB) and binds with increased affinity to serotonin-specific 5-HT2A receptors, located mainly in the prefrontal cortex, anterior cingulate, and parietal cortex [35,36]. As illustrated in Figure 2, the mechanism of action correlated with the path of psilocybin in the body consists of the following stages:

(1) After ingestion in natural or synthetic form, psilocybin is transported to the gastrointestinal tract;
(2) In the small intestine, under the action of alkaline phosphatase, psilocybin is enzymatically dephosphorylated, resulting in the active metabolite psilocin, which is pharmacologically responsible for the effects on the central nervous system (CNS) [8];
(3) Psilocin is absorbed at the enteric level and enters the systemic circulation, reaching therapeutically effective concentrations [37];
(4) Due to its lipophilic structure, psilocin easily crosses the BBB, reaching the CNS [34];
(5) At the synaptic level, it acts as a partial agonist of serotonergic 5-HT_2_A receptors, triggering profound changes [36];
(6) At the same time, psilocin is metabolised in the liver, mainly by cytochrome P450 (CYP450) enzymes into inactive metabolites [8];
(7) Excretion occurs via the kidneys and bile, in the form of active and inactive metabolites [31].

In the central nervous system, activation of 5-HT_2_A receptors causes a series of neurobiological effects documented in the literature:

- Stimulation of neuroplasticity through activation of neurotrophic gene transcription and increased expression of BDNF (brain-derived neurotrophic factor) protein [16];
- Desynchronization of the Default Mode Network (DMN), associated with reduced rumination and negative self-referentiality [38];
- Reorganisation of functional neural connectivity, which supports better cognitive flexibility and emotional integration [39];
- Stimulation of dendritogenesis and the formation of functional synapses [40];
- Facilitation of memory reconsolidation, an important process in post-traumatic disorder [19];
- Induction of the phenomenon of ego dissolution, a phenomenon associated with profound restructuring of self-perception [20].

Psilocybin has demonstrated a favourable therapeutic profile in multiple clinical studies. Among the most important positive effects are a significant and rapid reduction in symptoms of depression and anxiety, improvement of symptoms in substance uses disorders (alcohol, nicotine), sustained remission effects at 6–12 months post-intervention, improved functional brain connectivity, and increased emotional regulation capacity.

In terms of risks, the most common adverse reactions include acute anxiety (dysphoria) during the experience, temporary confusion, disorganised thinking, and temporary exacerbation of psychotic symptoms in patients predisposed to such events due to the nature of their existing psychopathologies.

Psychological effects include states of deep introspection and emotional reconnection, phenomena that contribute to the reconfiguration of maladaptive affective processes [40]. Despite these remarkable results, the exact mechanisms by which these acute changes generate long-lasting clinical benefits remain unknown. There is also insufficient data on the bioavailability of the active substance, long-term cumulative effects, pharmacological interactions, and risks in populations with neuropsychiatric comorbidities.

Preclinical and clinical studies suggest that the beneficial effects of psilocybin depend not only on molecular interactions but also on the context in which the substance is administered, known as the “set and setting” framework. The use of psilocybin must be strictly controlled in a clinical setting, with a prior diagnosis and psychotherapeutic support before, during, and after administration to ensure the safety and efficacy of treatment [12,41].

## 4. Quantitative Analysis of Clinical Studies on Psilocybin

### 4.1. Number of Clinical Studies Registered in the US and EU: Geographical Distribution

Over the past two decades, the number of clinical studies examining the efficacy and effects of psilocybin has increased rapidly. This exponential growth is particularly noticeable in the US compared to the EU. This analysis has identified differences in volume, geographical distribution, progress, and therapeutic objectives, reflecting not only distinct clinical priorities but also divergent socio-cultural and regulatory factors. This quantitative study aims to highlight the different characteristics of clinical trials, based on the total number of clinical trials registered in each region of interest, their distribution, target diseases, and the phase of development. It also compares the levels of development for each area and identifies the number of trials that are complete, active, or have not been authorised by the regulatory authority.

#### 4.1.1. Total Number of Studies and Participants: Age of Participants

**Figure 3:**
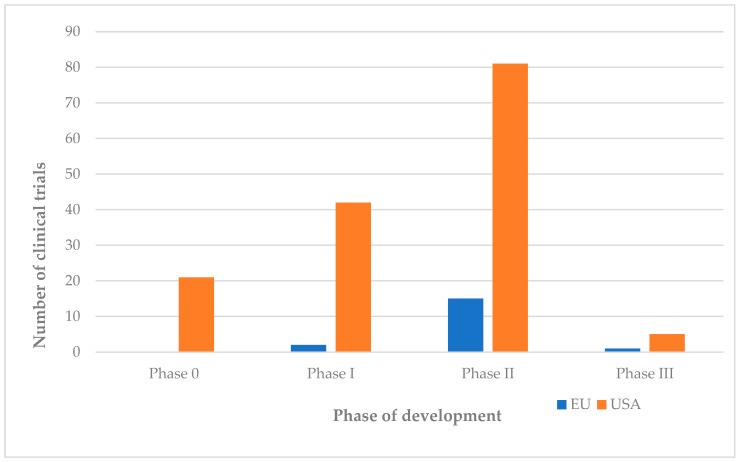
Distribution of clinical trials by development phase (from phase 0 to phase III). Description: Bar chart comparing the number of clinical trials in the US and the EU by development phase. Data source: ClinicalTrials.gov and CTIS; snapshot: 31 May 2025.

According to data collected from ClinicalTrials.gov and the EU Clinical Trials Register, as of 31 May 2025, 149 clinical trials involving psilocybin will be registered in the US, compared to only 18 trials in the European Union (Figure 3). Multicentre trials spanning the United States and the European Unions were treated as a single trial in aggregate counts; site-level data were used for geographical summaries. The significant discrepancy identified between the two regions may be associated with numerous factors, including research infrastructure, which has developed more extensively in the specific areas of the US, institutional support, and a favourable regulatory framework.

According to quantitative analysis estimates, more than 6000 volunteers have been and are being recruited in the 149 clinical trials identified on the ClinicalTrials.gov portal for the US.

On the other hand, in European studies, 1800 subjects were included in clinical trials focused on psilocybin. This disproportion is relevant not only statistically, but also from the perspective of external validation of the results obtained. Figure 3 shows that the distribution of clinical trials by development phase favours Phase II in both regions of interest.

This result suggests a strong focus on the efficacy and safety of psilocybin therapies. Moreover, there is a notable presence of Phase 0 studies in the US, which indicates a more permissive approval ecosystem, given that these studies are designed and approved in the vast majority of cases in tandem with Phase I and II studies.

The EU shows a more conservative progression in authorising psychedelic trials, driven by coordinated assessment under Regulation (EU) No 536/2014 via CTIS, with authorisation decision issued by the member states. Furthermore, the heterogeneity across member states in ethics review and controlled-substance accounts for the comparatively conservative approach in this region. European authorities therefore favour a process of exhaustive evaluation and verification before approval, rather than reactive post-introduction regulation. In the United States, initiation and progression of trials occurs under an Investigational New drug application (IND) in effect with an Institutional Review Board (IRB), while programs such as Fast Track and Breakthrough Therapy, increase the FDA interactions and facilitate movement towards phase II and III and speed up the development of the clinical trials.

**Figure 4:**
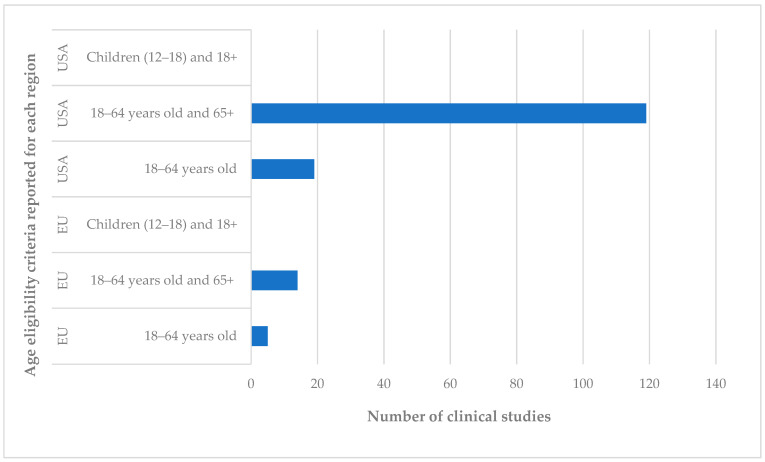
Distribution of clinical trials reported by participant age for each region (US and EU). Description: The graph compares the number of clinical trials by age group in the US versus the EU, highlighting a much higher number of trials for adults in the US. Data source: ClinicalTrials.gov and CTIS; snapshot: 31 May 2025.

The chart in Figure 4 shows the distribution of psilocybin clinical trials by participant age, analysing the US and EU regions separately. This classification facilitates a deeper understanding of the extent of demographic inclusion in current trials and provides insights into potential avenues of interest and regulatory focus for each region studied.

Upon reviewing the available data, it is worth noting that the majority of clinical trials are focused on adults aged 18 to 64 and seniors aged 65 and older. In the US, of the 138 clinical studies which have reached or passed the recruitment phase, 119 studies include these two age categories, 18 to 64 years old and 65+, which reflects the research focus on conditions that generally affect the adult population, such as substance use, anxiety disorders, depression, and PTSD. This approach is also supported by FDA policy, which recommends including comprehensive age categories in clinical trials to improve the validity of results [42].

In comparison, in the EU, the number of studies that include the 18- to 64-year-old and 65+ age groups is significantly lower. This result is explained by both the overall low number of clinical studies and the much more restrictive regulations regarding the study of psilocybin.

Another relevant aspect is the presence of children in psilocybin studies, which can only be identified in a single clinical study conducted in the United Kingdom, the results of which have not yet been published at the time of this analysis. This study, under Clinical Trials ID: NCT06798636, had not started recruitment at the time of analysis. This highlights the direction and intention to investigate the therapeutic effects of psilocybin on the young population and explore its use for conditions such as depression and anxiety at an early age. For this review, although the United Kingdom is no longer part of the European Union, it was included in this evaluation due to its essential role in promoting clinical research on psilocybin. The United Kingdom is home to centres of excellence in this field, such as Imperial College London, which actively collaborates with academic institutions in Germany and the Netherlands, thereby contributing significantly to the development and expansion of scientific knowledge in this emerging sector. However, it should be noted that these studies are scarce and difficult to approve due to the ethical risks and strict regulations that protect minors. In the EU and the US, this category is missing, confirming the conservative and restrictive orientation regarding clinical trials on children and young adults.

**Figure 5:**
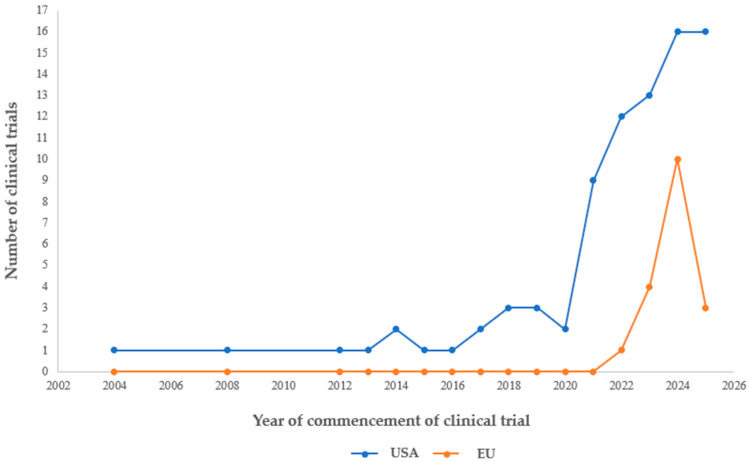
Evolution of clinical trials with psilocybin—comparison between the US and the EU. Description: The chart visually compares the number of clinical trials initiated in the US and the EU by year. Data source: ClinicalTrials.gov and CTIS; snapshot: 31 May 2025.

Based on the illustrated results, there has been a consistent increase in the number of clinical trials focusing on psilocybin in the US since 2010, followed by exponential growth after 2018 (Figure 5). This trend correlates directly with legislative changes at the federal and state levels, with examples in Oregon and Colorado, as well as the emergence of centres specialising in the study of psychedelic substances such as the Johns Hopkins Centre for Psychedelics. However, the key moment was the FDA’s decision to grant psilocybin “breakthrough therapy” status for treatment-resistant depression in 2018 and for major depressive disorder in 2019 [43]. In comparison, the EU has seen a more modest but noticeable increase in recent years (2022–2024), particularly in countries where the legislative frameworks have been adapted to allow for such research, such as Germany, the Czech Republic, Denmark, and the Netherlands.

#### 4.1.2. Geographic Distribution

**Figure 6:**
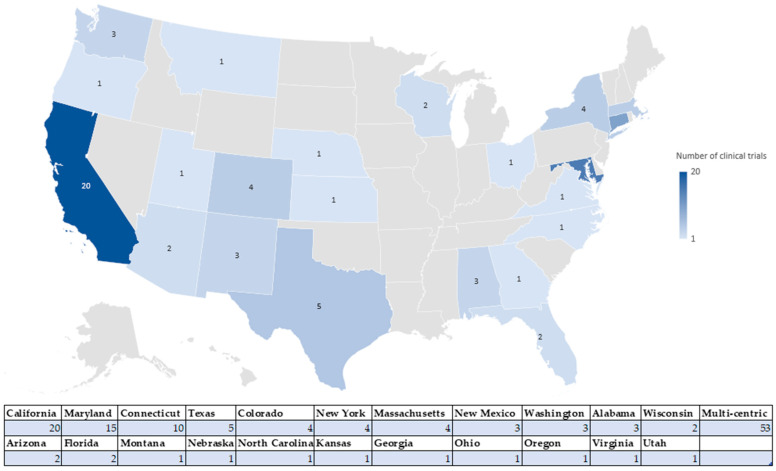
Map of the US: distribution of clinical trials by state (data May 2025). Description: States are coloured according to the number of trials, from 1 to 20 (California—20, Maryland—15, Connecticut—10, Texas—5, etc.), and 53 trials are multicentre. Data source: ClinicalTrials.gov and CTIS; snapshot: 31 May 2025, site counts inform geographic displays.

**Figure 7:**
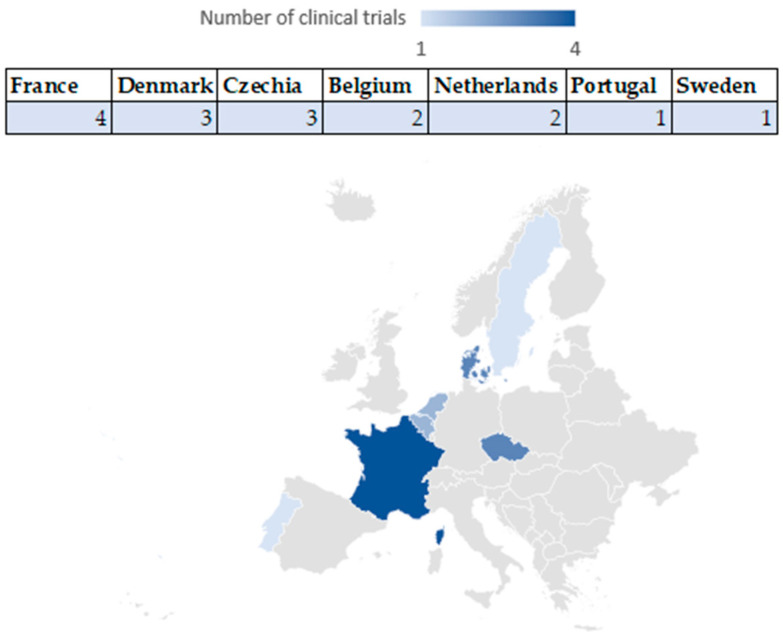
EU map: distribution of clinical trials by country (May 2025). Description: Countries are coloured according to the number of trials, from 1 to 4 (France—4; Denmark—3; Czechia—3; Belgium—2; Czech Republic and Denmark, 5; France and the Netherlands, 4; Sweden, 3; Belgium, 2; and the rest, 1. Data source: ClinicalTrials.gov and CTIS; snapshot: 31 May 2025.

The geographic distribution charts (Figure 6 and Figure 7) show that most clinical trials in the US are concentrated in states such as California (20 clinical trials), Maryland (15 clinical trials), Connecticut (10 clinical trials), Texas (with five clinical trials), and New York (with four clinical trials), Massachusetts (four clinical trials), New Mexico (with three clinical trials), Washington (with three clinical trials), Alabama (three clinical trials), followed by Wisconsin, and Arizona (with two clinical trials) and Florida, Montana, Nebraska, North Carolina, Kansas, Georgia, Ohio, Oregon, Virginia, Utah (with one clinical trial each). At the same time, 53 multicentre clinical trials were identified, with participants in California, New York, and Washington.

In the EU, the most active countries are France (with four clinical trials), Denmark (with three clinical trials), and Czechia (with three clinical trials), followed by Belgium and the Netherlands (with two clinical trials each), and Portugal and Sweden (with one clinical trial each). This regional concentration is directly related to the existence of centres of academic excellence and favourable regulations.

In parallel with psilocybin, studies frequently investigate other psychedelic substances such as LSD and MDMA. These are included in clinical protocols for conditions such as PTSD, addiction, or affective disorders, contributing to a broader understanding of the therapeutic potential of psychedelic substances [1].

### 4.2. Types of Conditions Targeted in Identified Clinical Trials

**Figure 8:**
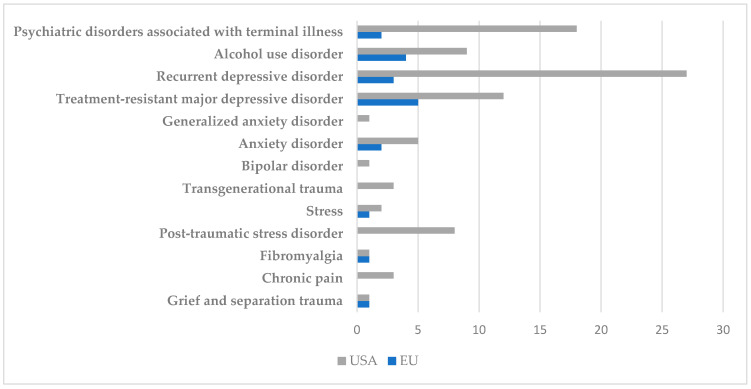
Distribution of studies by type of condition, US vs. EU. Description: Bar chart comparing the number of studies per condition, highlighting the priorities of each region. Data source: ClinicalTrials.gov and CTIS; snapshot: 31 May 2025.

Another central element of this quantitative analysis is the identification and comparison of the types of conditions for which psilocybin is being clinically investigated. This subchapter presents the differences and similarities between the US and the EU in terms of therapeutic priorities (Figure 8).

#### 4.2.1. Affective Disorders

In the US, psilocybin is predominantly used in studies focusing on the treatment of recurrent major depression and treatment-resistant depression. Other common indications include generalised anxiety and anxiety associated with terminal illness.

In the EU, studies focus on anxiety and depression associated with terminal illness diagnoses and depression related to palliative care.

These differences highlight distinct systemic orientations: in the US, the emphasis is on intervention in chronic disorders, whereas in the EU, the approach is based on the concept of ethics in palliative care.

#### 4.2.2. PTSD, Trauma, and Stress

PTSD is another significant indication, found mainly in the US, where the veteran and violence victim populations are actively included in research. In the EU, the approach is more modest, with studies addressing general psychological trauma. At the same time, chronic stress and adjustment disorders are conditions investigated in both regions, but with a higher incidence in the US.

#### 4.2.3. Addictions and Eating Disorders

Unlike the EU, research institutions in the US are pioneers in the study and investigation of psilocybin for the treatment of addiction. At this level, studies focus on alcoholism and smoking, where preliminary results are promising and show a reduction in use and relapse. At the same time, pilot studies have been proposed to investigate eating disorders and the effect of psilocybin on people diagnosed with anorexia nervosa. On the other hand, in the EU, this direction is in its infancy, and no clinical studies have yet been identified in this regard.

### 4.3. Comparison of Clinical Trial Phases

The distribution of trials across clinical phases is an essential indicator of the maturity and progress of medical research. This section details how psilocybin trials are distributed across phases I–IV and how the US differs from the EU in this regard.

**Figure 9:**
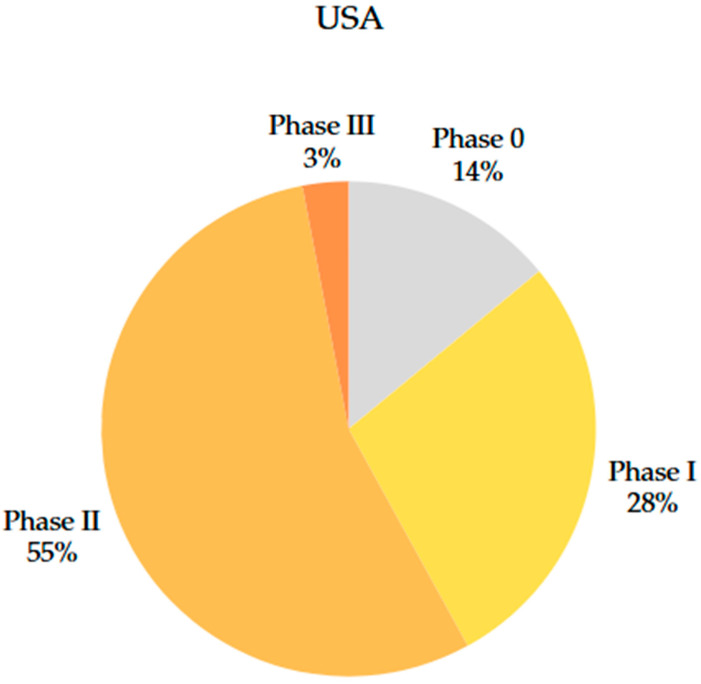
Percentage of the total number of studies identified for each development phase 0, I, II, III, in the US. Description: Pie chart showing the percentage of clinical studies by phase—0: 14%, I: 28%, II: 55%, III: 3%. Data source: ClinicalTrials.gov and CTIS; snapshot: 31 May 2025.

In the US, the distribution of clinical trials (Figure 9) is organised into phases 0, I, II, and III of development. Analysis of the data collected showed that there are 21 phase 0 studies (14% of the total), 42 phase I studies (28% of the total), 81 phase II studies (55% of the total), and 5 phase III studies (3% of the total) in the US.

**Figure 10:**
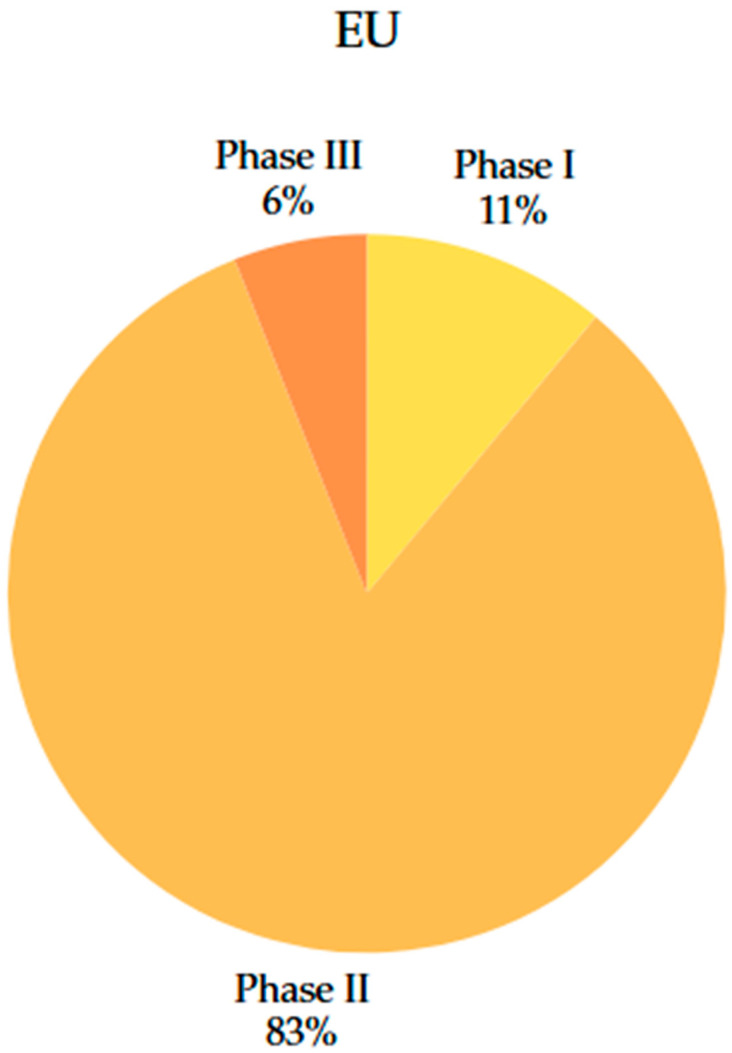
Percentage of the total number of studies identified for each development phase I, II, and III in the EU. Description: Pie chart showing the percentage of clinical studies by phase—I: 11%, II: 83%, III: 6%. Data source: ClinicalTrials.gov and CTIS; snapshot: 31 May 2025.

In the EU (Figure 10), only two phase I studies (11% of the total), 15 phase II studies (83% of the total), and a single phase III study were identified.

This contrast suggests faster progress in research in the US, but also a potentially increased risk associated with the rapid approval of studies, especially concerning participant safety. At the same time, it reiterates that progress in studies is slow-paced deliberately in the EU, and the legislative framework is more restrictive, focusing more on the efficacy of therapy and participant safety.

One cause identified by researchers in the field is national approvals, which involve multiple regulatory steps carried out over an extended period and are structured at both the European and national levels. This system differs from that in the US, where decisions are more dynamic in terms of federal regulations [32,42].

These variations are also supported by recent analyses included in The Lancet Psychiatry (2024), which explain that fragmented EU legislation supports a different dynamic and slower progress of clinical trials in psychiatry compared to the US. Heterogeneous requirements and the lack of comprehensive operational guidelines at the national level can also slow down approval procedures.

The category “recruitment completed, study ongoing” is only present in the EU, suggesting active research is underway. At the same time, the most significant proportion of studies identified in both the EU and the US have “active recruitment” status, highlighting the recent surge of interest in psilocybin. This exponential growth is also due to the emergence of specialized research centres such as those at the National Institute of Mental Health in the Czech Republic, the University of Copenhagen—NRU—Denmark, Imperial College London—Centre for Psychedelic Research—United Kingdom (although not a member state, it strongly influences research in this field) and international research networks such as PAREA (Psychedelic Access and Research European Alliance) with centres in several European countries and the OPEN Foundation in the Netherlands.

Furthermore, when it comes to completing studies, the US is again in first place, with 33 (23.74%) completed phase I and II studies, while no completed studies were identified in the EU. This difference is also supported by the considerable lead the US has in developing this area of research. As identified in a previous subchapter, in the EU, studies on psychedelic substances with volunteers were approved after 2021, compared to the US, where studies of this type have been supported since 2016 [9].

Another fact that supports the premise regarding the complexity of the approval and regulatory system in the EU is the “unauthorised” status identified exclusively in European studies [44].

In the US, only studies with the statuses “suspended” and “stopped before recruitment” were recorded, which may be related to financial and logistical issues; however, the possibility of stoppages for safety or legal reasons cannot be ruled out.

Finally, the status “unknown” appears only in connection with three studies in the US, and “recruiting by invitation” is reported in only one study, also in the US. This discordant note suggests possible non-compliance with transparency standards in US database records, or a focus on a narrow population, such as PTSD studies for war veterans.

## 5. Qualitative Analysis of Clinical Studies on Psilocybin

Clinical studies are an essential component in researching and evaluating the safety and efficacy of various therapies. These studies are divided into multiple phases, each with a distinct role and objective, which progressively contribute to the final approval of a treatment.

### 5.1. Clinical Trial Phases: Characteristics, Conduct, and Examples

Phase 0, although mainly found in the US, is a preclinical stage in which the pharmacokinetics and pharmacodynamics of the reference substance are evaluated simultaneously in minimal doses. This stage is often an integral part of Phase I studies in the EU, as well as preclinical tests and investigations.

In Phase 0 research, human involvement is minimal and typically occurs only in accredited research centers, with tests performed on a small number of healthy volunteers (10–15 volunteers). In the case of psilocybin, this stage contributes to dose safety testing [45]. Phase I is defined by safety and tolerability.

This stage involves a larger group of healthy volunteers, ranging from 20 to 100 people, and aims to assess safety, tolerability, and potential adverse reactions, while also analysing the pharmacokinetic profile. The studies are conducted in accredited centres, such as the Johns Hopkins Centre for Psychedelic Research in the US, where studies on the safety of psilocybin at moderate and high doses have already been conducted [26]. Institutions such as Maastricht University in the Netherlands and Imperial College London in the UK often conduct studies that are integrated into complex research structures or in the form of combined Phase I and II or Phase II and III studies [46].

The next category is Phase II studies, which test the effectiveness of the therapy on small groups of patients diagnosed with a target condition (generally between 100 and 300 patients). This is the first stage in which the innovative treatment can be compared with standard treatment or a placebo. The design of this type of study is generally double-blind, randomised, and involves proof-of-concept structures. In the US, Compass Pathways conducted and funded such a study for the treatment of treatment-resistant depression, and the results were published in The New England Journal of Medicine in 2021. In Europe, the University of Zurich and other multicentre institutions have replicated this phase II study with the support of the EMA. At this stage of development, the involvement of clinical specialists, psychiatrists, ethics advisors, and statisticians is essential [19].

Large-scale research is expanded through Phase III studies, which focus on confirming the efficacy of the therapy in a heterogeneous population using rigorous and replicable methods. The number of participants generally exceeds 1000, and given that the study is typically multicentre, universities, clinics, and hospitals are involved. In the US, the Usona Institute initiated one of the five studies existing at the time of data collection. It is multicenter, involving institutions in several US states, and focuses on the use of psilocybin in the treatment of major depression. On the other hand, in the EU, only one phase III study has been identified, supported and partially funded by the same company, Compass Pathways. The study was launched in 2023 in the UK, the Czech Republic, Denmark, Germany, Portugal, Spain, and the Netherlands, countries that have enjoyed the support of the European Commission and the EMA [47]. The results of phase III studies are an essential component of applications for legislative change. They are also used in marketing authorisation applications for medicines and are strictly regulated by the FDA in the US and the EMA in the EU.

Finally, phase IV (post-marketing) clinical trials aim to monitor the long-term efficacy of the treatment, any rare adverse effects, and its use in real-world settings. In the US, these studies are integrated into REMS (Risk Evaluation and Mitigation Strategies) registries, and in the EU, they are centralised through the EudraVigilance system. The data collected at this stage may influence changes in dosage, concentrations, the extension of therapeutic indications, or even market withdrawal. Currently, there are no active Phase IV studies on psilocybin in any region; however, long-term plans are in place to develop them, particularly if national regulations are revised.

The differences between the US and the EU in the conduct of clinical trials are significant at all levels, including planning and development, as well as at the regulatory level. While in the US, the FDA encourages adaptive designs to save time and resources, at the European level, adaptive designs are subject to strict regulations and verification thresholds that must be met to validate protocol and dosage changes before they are implemented. This can lead to delays in the clinical investigation process due to the desire to confirm the safety of participants [48].

### 5.2. Legislative and Regulatory Framework: US vs. EU

At the legislative level, clinical trials are directly influenced by the regulations in force in the country where the research is approved and conducted. At the same time, the results of completed studies have the potential to directly and indirectly influence legislative changes within each country, thus laying the foundations for a favourable context for future research. Indeed, regulations governing studies on psychedelic substances have reached a moderate stage of development, particularly in the EU, where in recent years many countries have made legislative changes to allow scientific progress [32].

In the US, clinical trials are coordinated and approved by the FDA and monitored by IRBs. In the case of psychedelic substances, including psilocybin, the FDA began to develop more flexible approaches to reviewing clinical trial documentation shortly after psilocybin therapy was granted breakthrough therapy status in 2019 for treatment-resistant depression [43]. This new designation was hailed as a new beginning in the recognition of the therapeutic and versatile effects of psilocybin, creating the ideal space for accelerating research approvals.

The clinical trial development process in the US includes the following stages/elements:

− application for IND;
− protocol review by the IRB;
− supervision of phases 0–IV, as appropriate, with a focus on safety and efficacy;
− the possibility of Fast Track or Breakthrough Therapy designation for innovative therapies, which facilitates the documentation process and access to funding.

This structure allows for flexibility in study design, including adaptive models that can facilitate changes in dosage and concentrations used, as well as modes of applied research based on interim results [49].

In the EU, the approval process for a clinical trial involves two types of committees operating simultaneously and is subject to both European and national guidelines. At the European level, the EMA is the regulatory body that validates and approves research, but this cannot be carried out without national evaluation by the competent authorities in each country involved. In 2024, the EMA published its first official guidance document, which includes a section dedicated to psychedelic substances in the treatment of depressive and anxiety disorders [32].

The general steps for initiating a clinical trial are as follows:

− submission of a Clinical Trial Application (CTA) to the national authority (ANMDMR in Romania) and the bioethics committee;
− evaluation of the CTA by the bioethics committee and the competent authority;
− registration in the EU-CTR database;
− ensuring compliance with EU Regulation 536/2014 on clinical trials.

The procedures for evaluating and initiating such a clinical trial can take significantly longer than in the US because substances such as psilocybin are included in Schedule I, which contains prohibited substances with no recognised medical use [50].

One notable difference between the US and the EU in this context is the numbering of clinical trials. While standard numbering (I-IV) is used in the EU, phase 0 is also used in the US for microdosing and early pharmacokinetic studies.

Adaptive study design is more common in US studies, where regulatory authorities offer researchers greater flexibility for real-time adjustments, compared to the rigid approach in the EU [49].

The approval structure in the EU involves authorisation issued by the member state in addition to coordination of the clinical trials assessments under Regulation (EU) No 536/2014 using CTIS, while in the US, IND approval is centralised and subject to federal regulations, coordinated by the local IRB. This allows for rapid approval [1].

Cultural and ethical differences are another distinctive factor influencing the pace of approval and development of clinical trials. In Europe, the emphasis is on ethics and the protection of study participants, with safety standards being much more specific in this regard. On the other hand, in the US, the emphasis is on innovation and adaptation to study conditions, especially in the case of emergency or “breakthrough” therapies [50].

Government and private financial support for research is more robust and relatively easy to access in the US, where the large number of non-profit organisations, research institutions, and private investors creates a favourable environment for clinical trials of this type. In the EU, many projects are funded by public funds or academic partnerships, which limits both the number of initiatives and the number of participants in such clinical trials [51,52].

Regulatory frameworks exert a strong influence on both the quantity and design of psilocybin studies, creating potential biases in the emerging evidence base. In the US, FDA has demonstrated a notable willingness to engage with industry sponsors. A key example is the designation of psilocybin-assisted therapy for TRD by COMPASS Pathways in October 2018, and later, for major depressive disorder by the Usona Institute in November 2019 as Breakthrough Therapies [43]. These designations have successfully promoted translational focus in the US evidence base by attracting investment and enabling large, multicenter, late-phase trials with regulatory alignment.

By contrast, the European landscape remains relatively fragmented and conservative. Approvals must navigate multiple national competent authorities along with EMA oversight, often favouring academically led, early-phase studies. While comprehensive source coverage is limited, this conservative regulatory climate tends to shape study design toward methodological strictness, including active placebos and rigorous blinding, over scale [53].

### 5.3. Comparative Study: Relevant Clinical Cases: US vs. EU

To better understand the differences between the clinical trial systems for psilocybin in the US and the EU, two representative studies from each region investigating the use of psilocybin for treating treatment-resistant depression (TRD) were analysed in parallel.

Among the most important clinical studies focusing on psilocybin in the treatment of depression is a survey conducted by Compass Pathways in the US, which was initiated in 2018 and partially completed (Phase II) in 2022. This phase II and III observational study, registered under number NCT03775200 on ClinicalTrial.gov, had over 200 participants suffering from treatment-resistant depression and was conducted in 10 specialised centres in the US, Canada, and the UK. Its results were published in the prestigious journal The New England Journal of Medicine [19].

The characteristics of the NCT03775200 study include:

- Design: phase II, double-blind study, three patient groups (25 mg psilocybin, 10 mg psilocybin, 1 mg psilocybin); psilocybin was administered in a single dose, and the total number of participants was 233; the evaluation was conducted over six weeks, using the standardised MADRS scale to compare symptoms;
- Sponsor: COMPASS Pathways, USA;
- Design features: adaptive, sample size (from 180 to 233) and maximum concentration dose (from 20 mg to 25 mg) were increased with FDA approval after analysis of interim results; the study also included participant training sessions, integration, and psychological support, following FDA protocols.
- Target population: patients with TRD who have previously failed at least two antidepressant treatments;
- Centres: US, Canada, UK;
- Results: significant reduction in depressive symptoms in the 25 mg group compared to the control group at 3 weeks post-single dose;
- Status: Phase II completed, actively recruiting for Phase III.

Another study of significant importance is that of the Central Institute of Mental Health in Germany. The study, under the code EudraCT 2019-003984-24, was designed in 2019 and approved in 2021. This study looked at 60 patients with significant, treatment-resistant depression and was conducted in a single research centre. As of 31 May 2025, the characteristics of the EudraCT 2019-003984-24 study include:

- Design: phase II, randomised, double-blind, active placebo-controlled (niacin) study with parallel groups; psilocybin was administered in a single dose of 25 mg and, for comparison, a control dose of 250 mg niacin; assessment was conducted over 4 weeks after the single dose, using the standardised MADRS scale to compare depressive symptoms;
- Sponsor: Central Institute of Mental Health, Germany;
- Design features: use of an active placebo, strict supervision by German regulatory authorities, BfArM and European authorities, EMA;
- Target population: adult patients (18–64 years) suffering from treatment-resistant depression who had a history of at least two previous failed pharmacological treatments;
- Centre: single centre, conducted exclusively in Germany, Mannheim;
- Results: final results have not yet been published;
- Status: study completed, with no reports of withdrawals, suspensions, or significant events during the study.

A critical strength of the COMPASS Pathways Phase IIb study (NCT03775200) lies in its scale and design. With 233 participants enrolled across multiple international sites, it remains the largest randomised controlled trial of psilocybin in TRD to date. The adaptive trial features, including sample size re-estimation and dose adjustment with FDA approval, highlight methodological rigour and responsiveness to interim data. The use of standardised outcome measures (MADRS), combined with structured preparation, integration, and psychological support, ensures comparability with conventional psychiatric trials and adherence to regulatory expectations. However, the study’s limitations should also be acknowledged. The reliance on a very low psilocybin dose (1 mg) as the control arm has been criticised as a potentially inadequate comparator. Additionally, the relatively short follow-up window (6 weeks) provides limited insight into the durability of therapeutic benefit, while industry funding has sparked debates over conflicts of interest and whether results may be applied outside of a strictly regulated clinical setting [19,54].

In contrast, the Central Institute of Mental Health trial (EudraCT 2019-003984-24) exemplifies a more academic, methodologically conservative approach. Its use of an active placebo (niacin 250 mg) is an important strength, as it better addresses the risk of unblinding by producing transient somatic effects. The trial was conducted under strict oversight by German and European regulators (BfArM, EMA), further reinforcing methodological robustness. Nevertheless, the study has notable limitations. Its smaller sample size (N = 60) and single-centre design limit both statistical power and external validity. Compared to the COMPASS trial, this study may provide stronger internal validity through its placebo design but at the expense of generalisation and scale, underlining a trade-off between methodological conservatism and translational reach.

Together, these trials highlight complementary contributions to the evidence base. The COMPASS study demonstrates scalability, international feasibility, and potential regulatory alignment for late-phase development, while the European trial underscores the importance of methodological stringency in ensuring regulatory robustness. A critical synthesis of both approaches suggests that future psilocybin trials would benefit from combining large-scale, multi-site recruitment with improved blinding strategies and longer follow-up periods to conclusively establish clinical efficacy and safety in TRD.

### 5.4. Factors Determining Differences Between the US and the EU in the Field of Clinical Trials with Psilocybin

The differences identified in the previous chapter in terms of the number of clinical trials, their distribution, and their progress can be explained by analysing the contextual and structural factors that directly influence the development of biomedical research. Three of the most important influencing factors are: government support and public policy, public attitudes toward this therapy, and industry involvement.

Firstly, government support is profoundly different between the two regions. At the same time, in the US, regulatory authorities are centralised at the federal level, in the EU, they are fragmented and divided between Member States and European institutions. This leads to variation in access to such studies and a longer time lag between the design and approval of a research project and its actual start.

Moreover, public attitudes toward the use of psychedelic substances directly influence the pace at which regulations can be implemented at the local level. In the US, there is greater openness to the concept of therapeutic psychedelic substances, which is also reflected in public policy. In the EU, the idea does not enjoy the same popularity, given that in the vast majority of European countries, psilocybin is classified as a high-risk drug, and the European cultural and historical context leads to more reserved perceptions in this regard.

Industry involvement is another key factor driving faster progress in the US compared to the EU. US biotech companies already possess the necessary infrastructure, capital, and motivation to invest in new and innovative therapies, as reflected in the numerous multicentre studies conducted across the country. In contrast, restrictive legislation in Europe does not favour private investment in this field, which is why most existing studies are publicly funded. These differences partly explain the gap between the two regions, considering the total number of clinical trials involving psilocybin, their progress, and their popularity in psychopathology research.

#### 5.4.1. Government Support and Public Health Policies

In the US, the FDA took a significant step in 2019 by designating psilocybin as a “breakthrough therapy” for treatment-resistant depression. This decision was made after analysing the results of two clinical trials, one conducted by Compass Pathways and the other by the Usona Institute. This event accelerated the evaluation and support process for studies involving psilocybin, initiating an ongoing dialogue between clinical trial sponsors and the FDA to enhance trial designs and ensure patient safety [13].

At the same time, the National Institutes of Health (NIHs) began offering grants dedicated to research on psychedelic substances, particularly for severe mental health disorders. Meanwhile, some US states have developed their progressive policies. Among the states that have implemented changes to public health policy in the US are:

− Oregon legalised the therapeutic use of psilocybin under regulated conditions, such as those included in clinical trials, in 2020 by adopting Measure 109; this led to the creation of a licensing system for the production and use of psilocybin [55];
− Colorado—followed a similar system to Oregon, adopting Proposition 122 in 2022, which allowed the use of psilocybin in clinical trials and for therapeutic use [56];
− Other states, such as California and Washington, developed pilot projects in 2023 to facilitate legislative changes to allow the development of Phase III and IV clinical trials for psilocybin [57].

In the EU, the central regulatory framework underwent changes in 2022 with the implementation of Regulation (EU) 536/2014, which permits the use of psilocybin in clinical trials under specific conditions. EU member states are free to synchronise with this regulation and align and change their national laws. Countries such as Germany, the Netherlands, the Czech Republic, and Denmark have taken essential steps in this direction.

In the Netherlands, research involving psychedelic substances such as psilocybin is permitted under stringent conditions and is classified as research with controlled substances.

Similarly, Germany has accelerated the process of authorising clinical trials using psilocybin through collaborations with academic institutions and transnational research projects with centres in the Czech Republic and the United Kingdom [58].

In the Czech Republic, regulations permit the use of psilocybin for scientific purposes with special authorisation from national authorities. The State Institute for Drug Control (SUKL) has approved clinical trials in collaboration with university centres to assess safety in the treatment of depression since 2021 [59].

At the same time, Denmark has recently simplified the process of preparing and approving dossiers for clinical trials involving psilocybin by introducing a fast-track assessment procedure in collaboration with the Danish Medicines Agency (DMA) and its national bioethics committees [32,60].

#### 5.4.2. Public Attitude and Social Acceptance

Public attitude toward psychedelic substances has changed in recent decades and is a key factor in the progress of alternative therapies in the treatment of psychopathologies. This change has been fuelled by the positive results of completed clinical studies and the active involvement of the media, scientific communities, and social media channels in disseminating scientific information.

A defining moment was the adoption of Measure 109 in Oregon in 2022, which allowed the regulated therapeutic use of psilocybin in licensed facilities, making it the first US state to adopt such legislation (Psychedelic Science Review, 2023). Opinion polls conducted in the US show a considerable increase in public support for the use of psychedelics.

In the EU, public perception is more reserved, but also heterogeneous, with each country having a relatively distinct public perception compared to that found in the US. Countries such as Germany and the Netherlands, with a recent history of tolerance towards the use of psychoactive substances, are more open both in terms of national regulations and public support.

On the other hand, countries such as Romania, Poland, and Slovakia have a restrictive approach due to prohibitive policies, lack of information, and stigma associated with drug use. Thus, even though the European legislative framework allows for the development of studies with psychedelic substances under safe conditions, social pressure and cultural conservatism can become an obstacle to participation in and support for such research.

Public attitudes are an important contextual factor in shaping the trajectory of psychedelic research and the translation of clinical findings into policy. In the US, nationally representative surveys demonstrate a clear trend toward growing acceptance. A 2017 YouGov poll reported that 53% of U.S. adults support medical research into psychedelics and 63% would personally consider psychedelic-assisted therapy if proven safe [61]. Moreover, the Berkeley Psychedelics Survey (2023) likewise found that 61% of registered voters favour regulated therapeutic access, and 78% support easing research restrictions [62]. National prevalence data also reflect increased exposure: a 2024 RAND survey estimated that 12% of US adults (18+) have tried psilocybin, with 3.1% reporting past-year use [63].

By contrast, Europe’s empirical data on public attitudes remains more fragmented—but recent developments suggest shifting dynamics. The 2025 European Drug Report notes expanding public and clinical interest in therapeutic applications of psychedelics, accompanied by targeted funding: for instance, Czechia’s national Drug Action Plan (2023–2025) includes resources for psychedelic research in addiction treatment, while the EU’s Horizon Europe program allocated €6.5 million in early 2024 to psychedelic research in palliative care [64]. A cross-sectional 2024 survey of European psychiatrists (419 participants from 33 countries) found general openness toward psychedelics and psychedelic-assisted psychotherapy, particularly among younger, spiritually oriented clinicians with previous personal experience of psychedelics [65]. Meanwhile, the grassroots mobilization exemplified by the 2025 launch of the PsychedeliCare European Citizens’ Initiative (ECI), designed to gather one million signatures in support of regulated access and funding for psychedelic therapies, also signals growing public engagement [66].

Taken together, these data suggest that while the US currently exhibits broader societal acceptance and familiarity with psychedelics, Europe is experiencing a growing, though uneven, acceptance, driven by clinician receptivity, emerging public advocacy, and evolving policy frameworks.

#### 5.4.3. Involvement of the Pharmaceutical Industry and Private Investors

The scale and pace of clinical trials in the US and the EU are also influenced by the level of involvement of the pharmaceutical and biotechnology industries. From this perspective, private investment capital is crucial in developing emerging investments dedicated to this research sector.

In the US, the psychedelic research ecosystem is closely linked to the private sector. Companies such as Compass Pathways, Atai Life Sciences, Usona Institute, MindMed, and MAPS PBC have invested in the vast majority of clinical trials on such substances. They are leading the way in this category of research and innovation [67,68,69].

In addition to collaborating with internationally renowned academic institutions such as New York University and Johns Hopkins University, these companies also have a favourable infrastructure that gives researchers access to state-of-the-art laboratories, private clinics, data management platforms, and strategies for integrating digitally assisted therapies.

In the EU, research in this field is mainly dominated by academic centres and university hospitals. In particular, research and higher education institutions, such as Maastricht University (Netherlands), Charité Berlin (Germany), and the Psychiatric Research Institute (Switzerland), initiate and coordinate clinical studies, typically in the early stages. These are financially supported by research development grants, Horizon Europe, FP7, and primarily funded by the public budget [69].

In terms of financial involvement, private companies are limited by several factors, including restrictive national regulations and an unpredictable economic market. At the same time, very few private companies have investments in psychedelic substances in their portfolios, except for several companies in the UK, such as Beckley Psytech, which, although no longer in the EU, continues to report to general European regulations and standards. In this case, the strong American influence is gaining ground in the UK’s investment ecosystem, despite EU policies emphasising caution and conservatism [70].

The gap in the maturity of clinical studies between the two reference regions, the US and the EU, is thus generated by the multitude of factors presented. While the US is preparing for the commercial integration of psilocybin for large-scale use in the treatment of a wide range of psychopathologies, the EU is taking small steps toward advanced research focused on the safety and efficacy of therapies, with limited development and a range of restrictive national policies.

## 6. Challenges in Psilocybin Clinical Trials

Despite the resurgence of academic and clinical interest in the use of psilocybin in the treatment of psychopathologies and various neurodegenerative disorders, the development and approval of clinical trials remain a complex process that often encounters multiple obstacles. These vary from region to region but share legislative and ethical issues, investment and funding opportunities, methodology, and sometimes even the cultural climate [13]. This section reviews a significant set of barriers that slow or hinder the progress of clinical studies, with a focus on the legislative framework in the regions of interest.

Parallel investigations on other psychedelic substances have significantly influenced the development of psilocybin research infrastructures in the US and EU. In the US, a first FDA-approved Phase 1 trial of MDMA was initiated in 1994 and advanced to Phase 2 trials by the mid-2000s under the supervision of the Multidisciplinary Association for Psychedelic Studies (MAPS), with pivotal Phase 3 programs beginning in 2017 for the treatment of PTSD [71,72]. This multi-decadal trajectory established a practical blueprint for conducting trials with Schedule I substances, influencing psilocybin investigators in the design of therapist-assisted protocols and safety monitoring. Moreover, FDA has already granted “breakthrough therapy” designations for MDMA and LSD treatments. In Europe, renewed human studies with LSD at the University of Zurich and the Imperial College London psilocybin pilot for depression provided methodological continuity and institutional legitimacy. The formal launch of the Imperial Centre for Psychedelic Research in 2019 consolidated shared neuroimaging and psychometric infrastructure that directly supported psilocybin trials [73].

Besides these compounds, research on N,N-Dimethyltryptamine (DMT) and ayahuasca has also contributed to the broader ecosystem in ways that indirectly supported psilocybin research. Studies of DMT in controlled settings expanded regulatory familiarity with short-acting psychedelics and generated experience with acute safety protocols, while ayahuasca research in Spain and Brazil demonstrated the feasibility of integrating long-duration psychedelic sessions into Western clinical trial frameworks [74]. Investigations into ibogaine for addiction and mescaline in smaller academic contexts added further evidence of therapeutic potential, drawing philanthropic and commercial investment into the field as a whole [75,76]. Collectively, these parallel research efforts fostered a critical mass of infrastructure that facilitated the faster scaling of psilocybin research in the US and the EU than it could have done separately.

### 6.1. Legislative and Institutional Barriers and Infrastructure

One of the most common obstacles to the development of clinical trials focusing on psilocybin is the legal status of the substance. In most EU member states, as well as in many US states, psilocybin is still classified as a controlled substance found in Schedule I, being considered to have no recognised medical use and a high potential for abuse [77,78].

This classification limits access to the use and research of psilocybin by imposing a restrictive legislative framework, requiring special authorisations for its inclusion in clinical trials and extensive research. In many cases, the study of psilocybin is only permitted with special agreements and standard methodologies, which are often subject to change and require collaboration with institutions authorised to handle and process the substance.

In the US, a licensing program has been developed that authorises researchers to investigate and use classified psychedelic compounds, ensuring a standard of quality for the substances used and the safety of study participants [79].

On the other hand, in the EU, although psilocybin has been included in emerging directions for research into psychedelic substances for the treatment of severe mental disorders, the decentralised authorisation system, which includes different levels of approval at the national level, creates a lack of predictability for clinical trial initiators [68].

Moreover, beyond the capital and sponsorship, clinical trials development is gated by site-level and system-level infrastructure. Some of the key elements related to research infrastructure include: controlled-substances pharmacy capabilities (secure storage, chain-of-development, authorised staff), reliable investigational medicinal product supply, import and export logistics and purpose-built clinical environments for preparing and integrating psylocibin in clinical sessions. Furthermore, the clinical trials results rely on trained psychotherapy personnel operating under standardised protocols and supervision combined with data and safety monitoring boards.

In practice, these components are more commonly co-located within the US academic medical centres and specialised private clinics, which have had prior Schedule I research experience. In the EU, the determinant factors are more commonly found in leading university hospitals and research institutes. However, the heterogeneity across member states in narcotic licensing, ethics review requirements and supply chain management of investigational products can introduce additional coordination steps which set back the development of clinical trials on psylocibin.

### 6.2. Ethical Challenges and Social Acceptability

The use of psilocybin in clinical research raises numerous ethical questions, especially in the context of the historical stigmatisation of psychedelic substances. Ethics committees must rigorously assess the potential risks to participants, particularly concerning possible intense or exacerbated psychological reactions and possible complications of psychiatric symptoms [26].

Another key ethical issue is obtaining informed consent, ensuring that volunteers are fully aware of the implications of participating in such a study and the risks they are taking, including the nature of the experience and its emotional consequences. This is further complicated by the subjective nature of patients’ experiences with psilocybin, as their reactions are unpredictable and strongly influenced by their own beliefs, traumas, and past experiences [45].

From a related perspective, the lack of long-term information on the safety of psilocybin in therapy for vulnerable individuals, such as patients with severe mental illness or traumatic histories, adds a factor of reluctance to the approval process for these studies. At the same time, in many cultures, both Eastern and Western, psychedelic substances remain associated with recreational use, the stigma of substance abuse, and the counterculture era, which still negatively influence public perception and make it difficult for researchers to gain political support.

### 6.3. Financial and Methodological Barriers

The design and development of clinical trials require substantial capital as well as significant logistics and interdisciplinary coordination. In particular, research on psilocybin requires an appropriate framework to support studies, special infrastructure, qualified and trained personnel, expert therapists, safety systems, and post-study follow-up. The cost of all these implications is explained by the innovative potential of the substance, but cannot be guaranteed, especially if the substance is not legally patentable. This uncertainty reduces access to funding and ultimately investor interest, especially in the EU, where psilocybin is still classified as a controlled substance without exceptions for research in most countries, as is the case in many US states [13].

In the US, a significant portion of investment comes from non-profit organisations and biotechnology companies in states that have changed their legislation to relax restrictions on psilocybin research. However, competition for funding is high, and studies on substances such as psilocybin are often disadvantaged in ethical and financial evaluations.

Another significant obstacle is the difficulty of standardising the methodology and protocols for using psilocybin. The psychedelic experience associated with psilocybin use is deeply subjective and influenced by the context of administration, making it difficult to control variables in a classic randomised design [35].

Furthermore, the duration of the effects, the need for prior preparation and post-administration evaluation sessions over an extended period, as well as possible interactions between psilocybin, psilocin, and other drugs used in associated psychotherapy, pose unique challenges in designing studies on this substance.

At the same time, comparing clinical trial results from different centres, countries, and regions is challenging because the clinical trial system is difficult to replicate, and volunteers’ experiences are unique to their own [14].

### 6.4. Patient Safety Risks: Challenges in Scaling Therapy

Although psilocybin has a favourable safety profile when administered in a controlled setting, the risks should not be overlooked. The most common adverse effects associated with psilocybin ingestion include acute anxiety, paranoia, temporary confusion, nausea, and changes in perception of the environment [41,80].

In the same context, severe long-term adverse effects have not yet been studied in detail. Transient psychotic episodes have been reported, especially in patients with an unclear psychiatric history. In this context, there are concerns about the potential for reactivation of suppressed trauma, episodes of dissociation, and difficulty reintegrating into society after the experience. Furthermore, the lack of a standard training for therapists may lead to variations in how adverse effects are addressed by therapists across different studies. Therefore, the safety of volunteers is contingent upon the environment during the experience, rigorous screening and assessment of volunteers’ traumatic experiences, as well as continuous medical supervision and standardised specialised psychological assistance [13].

Another important aspect is the translation of successful clinical studies into specialised medical practice. Although the results of studies completed to date are promising, there is no harmonised legislative framework for the authorisation and therapeutic distribution of psilocybin. Furthermore, there is a need for specialised centres with trained therapists and precise protocols for administering psilocybin and managing its adverse effects. Without these regulatory frameworks to ensure the safety of psilocybin therapy, this innovative treatment may never move beyond the experimental context [35].

Thus, the risks and challenges associated with the use of psilocybin can only be overcome and managed through a structured and standardised approach to processes, training of specialised personnel, and expansion of clinical research. These measures can not only improve the intervention experience but also strengthen the confidence of the general public and the scientific community. Therefore, these steps may provide the necessary foundation for promoting and developing new large-scale research to enable this revolutionary therapy to help people affected by mental disorders that are resistant to conventional treatments [8,12].

Differences in trial phases across psilocybin studies have direct implications for clinical practice and policy. Early-phase work (Phase I–IIa) has been crucial for establishing safety, dosing, and feasibility but remains limited to selected populations and specialised research settings. In contrast, larger Phase IIb and Phase III trials provide the scale and methodological rigour needed to generate reliable effect estimates, detect rare adverse events, and support regulatory decisions. These late-stage studies are essential for translating psilocybin from experimental intervention into therapeutic practice, as reflected in regulatory milestones such as the FDA’s Breakthrough Therapy designation and ongoing EMA evaluations [80].

Clinically, this progression means that early findings should be interpreted as proof-of-concept, while Phase III outcomes will determine whether psilocybin can be integrated into treatment guidelines and reimbursement frameworks. Policy-wise, the US has moved more rapidly due to large industry-sponsored programs, whereas European adoption is likely to advance more cautiously, building on smaller but methodologically stringent academic trials. Together, these differences highlight how both exploratory studies and late-stage multicenter trials are complementary in shaping the clinical and policy scenery for potential psychedelic-assisted therapies.

## 7. Conclusions

This study highlights the significant differences between the United States and the European Union in the development of clinical trials involving psilocybin. The US dominates this field both quantitatively and qualitatively, with significantly more studies registered and approved, using advanced methodologies and an institutional approach that favours rapid innovation. In contrast, research in the EU is predominantly exploratory, conducted in a few academic centres, and the more restrictive and fragmented legislative framework contributes to the slow pace of progress. Legislative differences, availability of funding, and cultural attitudes toward research into psychedelic substances create a clear gap between the two regions, both in terms of clinical priorities and the types of pathologies investigated.

To support balanced and responsible development in the field of psilocybin-assisted therapies, reform of the EU’s regulatory framework is essential. This should include harmonisation of the guidelines issued by the EMA, facilitation of innovation pathway mechanisms, establishment of uniform ethical standards, and adequate training of specialised personnel. There is also a need for expanded international collaboration and more diversified funding to enable rigorous multicentre studies. The integration of psilocybin into public health systems could bring significant benefits, particularly in the context of the global mental health crisis, by offering an effective alternative for patients who are resistant to conventional treatments.

Research on psilocybin is currently at a turning point, and future directions depend on the cohesion between innovative policies, solid scientific evidence, and ethical commitment. Strengthening the scientific basis requires the development of longitudinal studies, safety testing in vulnerable populations, and the development of predictive models of individual response. Within a regulated and well-supported medical framework, psilocybin has the potential to become a valuable therapeutic tool, capable of contributing significantly to reducing the burden of mental illness and transforming the therapeutic paradigm in neuropsychiatry.

## Data Availability

No new data were created or analyzed in this study. Data sharing is not applicable to this article.

## Abbreviations

The following abbreviations are used in this manuscript:

AD	anxiety disorders
BBB	blood–brain barrier
BDNF CTIS	brain-derived neurotrophic factor Clinical Trials Information System
CTA	Clinical Trial Application
DMN	Default Mode Network
DMT	N,N-Dimethyltryptamine
EMA	European Medicines Agency
EU	European Union
EU CTR	EU Clinical Trials Register
EUDA	European Union Drugs Agency
FDA	Food and Drug Administration
IRB	Institutional Review Board
LSD	lysergic acid diethylamide
MAPS	Multidisciplinary Association for Psychedelic Studies
MD	major depression
MDMA	3,4-methylenedioxy-N-methylamphetamine
OCD	obsessive-compulsive disorders
PAREA	Psychedelics Access and Research European Alliance
PTSD	post-traumatic stress disorder
RDD	recurrent depressive disorder
TRD	treatment-resistant major depression
USA/US	United States of America
WHO	World Health Organisation
